# Supplementary material for: All-at-once RNA folding with 3D motif prediction framed by evolutionary information
Source: Nat Methods. 2025 Oct 3;22(10):2094–106. doi: 10.1038/s41592-025-02833-w (PMC12510878; doi:10.1038/s41592-025-02833-w)
Supplement: Supplementary file 3 — Source code to run CaCoFold-R3D. Data and results. [file 41592_2025_2833_MOESM3_ESM.gz › supplemental_material/results/CaCoFold-R3D/CaCoFold-R3D_Rfam_vshuffle/RF02128_GNAS-AS1_2.R2R.sto.pdf]

RF02128_GNAS-AS1_2

5′-● U ● ● A R Y G G U C U U C C U U C C A G G ● C Y Y ● U Y C C ○ U R G G A A A G G C G Y ● R C R G Y G Y ● C C ● R R A C U U A G Y U ● Y G Y ● G ○ ● C U G C R G C G C ○ ○ ○ ● ● A ● ● R Y ● ● Y U G G A Y G C Y ● Y Y ● U G G U C U U C ● C
